# Apolipoprotein E promotes white matter remodeling via the Dab1‐dependent pathway after traumatic brain injury

**DOI:** 10.1111/cns.13298

**Published:** 2020-03-01

**Authors:** Zhi‐Jian Huang, Fang Cao, Yue Wu, Jian‐Hua Peng, Jian‐Jun Zhong, Yong Jiang, Cheng Yin, Zong‐Duo Guo, Xiao‐Chuan Sun, Li Jiang, Chong‐Jie Cheng

**Affiliations:** ^1^ Department of Neurosurgery The First Affiliated Hospital of Chongqing Medical University Chongqing China; ^2^ Department of Cerebrovascular The Affiliated Hospital of Zunyi Medical College Zunyi China; ^3^ Department of Neurosurgery The Affiliated Hospital of Southwest Medical University Luzhou China; ^4^ Department of Neurosurgery Affiliated Hospital of the University of Electronic Science and Technology of China Sichuan Provincial People's Hospital Chengdu China

**Keywords:** apolipoprotein E, axonal regeneration, Disabled‐1, traumatic brain injury

## Abstract

**Introduction:**

Axonal injury results in long‐term neurological deficits in traumatic brain injury (TBI) patients. Apolipoprotein E (ApoE) has been reported to activate intracellular adaptor protein Disabled‐1 (Dab1) phosphorylation via its interaction with ApoE receptors. The Dab1 pathway acts as a regulator of axonal outgrowth and growth cone formation in the brain.

**Aims:**

We hypothesized that ApoE may alleviate axonal injury and regulate axonal regeneration via the Dab1 pathway after TBI.

**Results:**

In this study, we established a model of controlled cortical impact (CCI) to mimic TBI in vivo. Using diffusion tensor imaging to detect white matter integrity, we demonstrated that APOE‐deficient mice exhibited lower fractional anisotropy (FA) values than APOE^+/+^ mice at 28 days after injury. The expression levels of axonal regeneration and synapse plasticity biomarkers, including growth‐associated protein 43 (GAP43), postsynaptic density protein 95 (PSD‐95), and synaptophysin, were also lower in APOE‐deficient mice. In contrast, APOE deficiency exerted no effects on the levels of myelin basic protein (MBP) expression, oligodendrocyte number, or oligodendrocyte precursor cell number. Neurological severity score (NSS) and behavioral measurements in the rotarod, Morris water maze, and Y maze tests revealed that APOE deficiency caused worse neurological deficits in CCI mice. Furthermore, Dab1 activation downregulation by the ApoE receptor inhibitor receptor‐associated protein (RAP) or Dab1 shRNA lentivirus attenuated the beneficial effects of ApoE on FA values, GAP43, PSD‐95, and synaptophysin expression, and neurological function tests. Additionally, the effects of ApoE on axonal regeneration were further validated in vitro. In a mechanical scratch injury model of primary cultured neurons, recombinant ApoE protein treatment enhanced axonal outgrowth and growth cone formation in injured neurons; however, these effects were attenuated by Dab1 shRNA, consistent with the in vivo results.

**Conclusion:**

Collectively, these data suggest that ApoE promotes axonal regeneration partially through the Dab1 pathway, thereby contributing to functional recovery following TBI.

**Abbreviations**
ApoE: apolipoprotein E
APP: β‐amyloid precursor protein
CCI: controlled cortical impact
Cdc42: cell division control protein 42
Dab1: Disabled‐1
DTI: diffusion tensor imaging
FA: fractional anisotropy
OC: oligodendrocyte
OPC: oligodendrocyte precursor cell
TBI: traumatic brain injury

## 1 INTRODUCTION

Traumatic axonal injury (TAI) is involved in almost all forms of brain trauma, especially traumatic brain injury (TBI), which results in long‐term disability. TAI tends to disrupt the normal axoplasmic transport and electric signal transmission of axons, resulting in progressive axonal degeneration. However, axonal regeneration begins at some point after injury. Guided by the newly regenerated growth cone, residual axons spontaneously sprout from residual injured axonal tips and, therefore, potentiate the extension of axons to the destined area. However, the sprouting ability of injured neurons in the adult central nervous system (CNS) is very limited, accounting for the poor functional recovery after TBI.^1^ Axonal regeneration is essential for synapse formation and synaptic maintenance, both of which are essential for neuronal circuit formation and function and ultimately contribute to functional recovery. Hence, strategies to promote axonal regeneration may provide promising benefits for TBI therapies.

Apolipoprotein E (ApoE) is a plasma protein responsible for transporting lipids and cholesterol. Moreover, ApoE is pivotal in modulating CNS neuronal responses to injury.^2^ As the dominant apolipoprotein in the CNS, ApoE has been suggested to support a protective role of ApoE in TBI in many studies. For example, ApoE regulates neurogenesis, blood‐brain barrier (BBB) integrity, inflammatory responses, and cell apoptosis after TBI.^3^, ^4^, ^5^ Furthermore, neurite outgrowth in peripheral nerves is impaired in APOE‐deficient mice,^6^ while ApoE‐mimetic peptide promotes axonal regeneration and myelination after peripheral axonal injury.^7^ However, whether ApoE also contributes to axonal regeneration in CNS neural injury remains largely unknown.

ApoE contains two major structural domains: The N‐terminal domain contains the low‐density lipoprotein receptor (LDLR) binding region, and the C‐terminal domain contains the lipid‐binding region. By binding to its receptors in the neural cell membrane, ApoE activates several intracellular kinases, resulting in the phosphorylation of intracellular adaptor protein Disabled‐1 (Dab1).^8^, ^9^ Extensive evidence has revealed that ApoE receptors, especially the very low‐density lipoprotein receptor (VLDLR) and the apolipoprotein E receptor 2 (ApoER2), cluster upon ligand binding and recruit Dab1 to an NPxY motif in their cytoplasmic domains, which leads to the tyrosine phosphorylation of Dab1 by nonreceptor tyrosine kinases of the Src family.^10^ Various downstream signaling pathways of Dab1 connect ApoE to the actin and microtubule cytoskeleton. A central step in Dab1‐driven dendrite outgrowth and growth cone formation is facilitated by the activation of the Rho‐GTPase protein cell division control 42 (Cdc42).^11^ Cdc42 is a monomeric G protein that is switched on when bound to GTP and switched off when bound to GDP. Activated Cdc42 is pivotal in regulating the cytoskeleton dynamics of actin filaments and microtubules. Cdc42 promotes actin accumulation in the growth cone to potentiate axon elongation.^12^ The abovementioned evidence supports the activation of the Dab1 pathway as a therapeutic strategy in axonal regeneration. However, the effect of the Dab1‐Cdc42 pathway in TBI needs to be further clarified. In the current study, we hypothesized that ApoE may promote axonal regeneration following TBI via a Dab1‐dependent pathway.

## 2 MATERIALS AND METHODS

APOE‐deficient adult male mice (APOE^−/−^, purchased from Peking University Laboratory Animal Centre) and wild‐type C57BL/6 adult male mice (APOE^+/+^, purchased from Laboratory Animal Centre at Chongqing Medical University) weighing 25‐30 g were utilized in this study. The genetic background of APOE^−/−^ mice is the same as that of C57BL/6 mice. All animal procedures were approved and supervised by the Animal Ethics Committee of Chongqing Medical University. All studies involving animals are in accordance with the ARRIVE guidelines for reporting experiments involving animals.

### 2.1 Controlled cortical impact (CCI) model

The CCI model was produced by the TBI‐0310 TBI Model system (Precision Systems and Instrumentation, USA), as previously described.^13^ Each mouse was anesthetized with an induction gas mixture (3% isoflurane with 1 L/min 100% oxygen). Once the mouse was fully sedated (steady breath rate coupled with the absence of toe‐pinch reflex), the mouse was removed from the induction chamber, placed abdomen‐down on a heating pad (37℃), and administered a maintenance gas mixture (1.5% isoflurane (range: 0.9%‐1.8%) with 0.5 L/min 100% oxygen) for the remainder of the surgery. Briefly, a 5‐mm left lateral craniotomy centered at 1.0 mm lateral to the midline and 3.0 mm anterior to lambda was performed. The CCI injury was produced using a pneumatic cylinder with a 3‐mm diameter flat‐tip impounder at an impact velocity of 6.0 m/s, a dwell time of 40 ms, and a cortical contusion depth of 0.6 mm. The body temperature of each mouse was maintained at 36‐37°C throughout the duration of the surgery.

### 2.2 Construction of the Dab1 shRNA lentivirus

Small interfering RNAs targeting the mouse Dab1 gene were designed by Shanghai GeneChem Co., Ltd., China. The optimal small interfering RNA sequence against mouse Dab1 (shRNA1: 5′‐AGTGTGAACAAGCTGTGTA‐3′, shRNA2: 5′‐TCAGCATCACCATGCTGTT‐3′) was then cloned into the plasmid pGCL‐GFP, which encodes a human immunodeficiency virus (HIV)‐derived lentiviral vector containing a multiple cloning site for the insertion of shRNA constructs to be driven by an upstream U6 promoter and a downstream cytomegalovirus promoter‐GFP (marker gene) cassette flanked by loxP sites. The nonsilencing (NS) shRNA was constructed by a similar process (5′‐TTCTCCGAACGTGTCACGT‐3′). These modified plasmids were further cotransfected into HEK293T cells with lentiviral packaging plasmids to generate a Dab1 shRNA‐expressing lentivirus or a control shRNA‐expressing lentivirus.

At 6 days prior to CCI, Dab1 shRNA lentivirus was used to inhibit Dab1 expression, while a control shRNA‐expressing lentivirus (NC shRNA) was used as a control. Both of these viruses were diluted to 1.3 × 10^10^ pfu/mL before use. After appropriate anesthesia, a total volume of 4 µL of lentivirus solution was stereotactically injected into the right lateral ventricle (1 mm lateral to and 0.5 mm posterior to bregma with a depth of 2.0 mm).

Receptor‐associated protein (RAP) is a strong competitive blocker that inhibits the binding of ApoE to its receptors. RAP (RayBiotech) was dissolved in 0.9% (w/v) saline. Two micrograms of RAP was intracerebroventricularly injected in a total volume of 3 µl at 1‐3 days postinjury. ML141 (synonym: CID‐2950007) is a potent, selective and reversible noncompetitive inhibitor of GTP‐Cdc42 with low micromolar potency and selectivity against other members of the Rho family of GTPases. ML141 (Selleckchem) at 10 mg/kg body weight was dissolved in dimethylsulfoxide (DMSO) and was intraperitoneally injected at 1‐7 days postinjury.

### 2.3 Diffusion tensor imaging (DTI)

DTI was used to detect axonal regeneration in the corpus callosum (CC) after CCI. All magnetic resonance imaging (MRI) scans were performed on a Bruker 7T (70/20) system (Bruker Biospin). Mice were imaged before injury using DTI so that each mouse could serve as its own control. Each mouse was anesthetized and mounted on a Bruker animal bed, and body temperature was maintained at 37°C with respiratory rate continuously monitored. DTI images were acquired with a single‐shot spin‐echo echo‐planar imaging (EPI) sequence in the coronal plane. Diffusion‐sensitive gradients were applied in 30 noncollinear directions at b = 1000 s/mm^2^. Five additional images at b = 0 s/mm^2^ were also acquired. The acquisition parameters were field of view (FOV)= 2.5 × 2.5 cm^2^ at a matrix resolution of 128 × 128, repetition time (TR)/echo time (TE)=3000/25 ms, slice thickness = 0.5 mm for a total of 15 slices, and two averages, covering the same area as the coronal structural acquisitions. The diffusion tensor eigenvalues (λ1, λ2, λ3) were computed using the ParaVision 6.0 (Bruker BioSpin) diffusion tensor calculation module. The fractional anisotropy (FA) was calculated using the following equation:

$$\text{FA}=\surd 1/2\left(\surd \;\left({\left(\lambda 1-\lambda 2\right)}^{2}+{\left(\lambda 1-\lambda 3\right)}^{2}+{\left(\lambda 2-\lambda 3\right)}^{2}\right)/\surd \;\left({\lambda 1}^{2}+{\lambda 2}^{2}+{\lambda 3}^{2}\right)\right)$$

**Figure 1:**
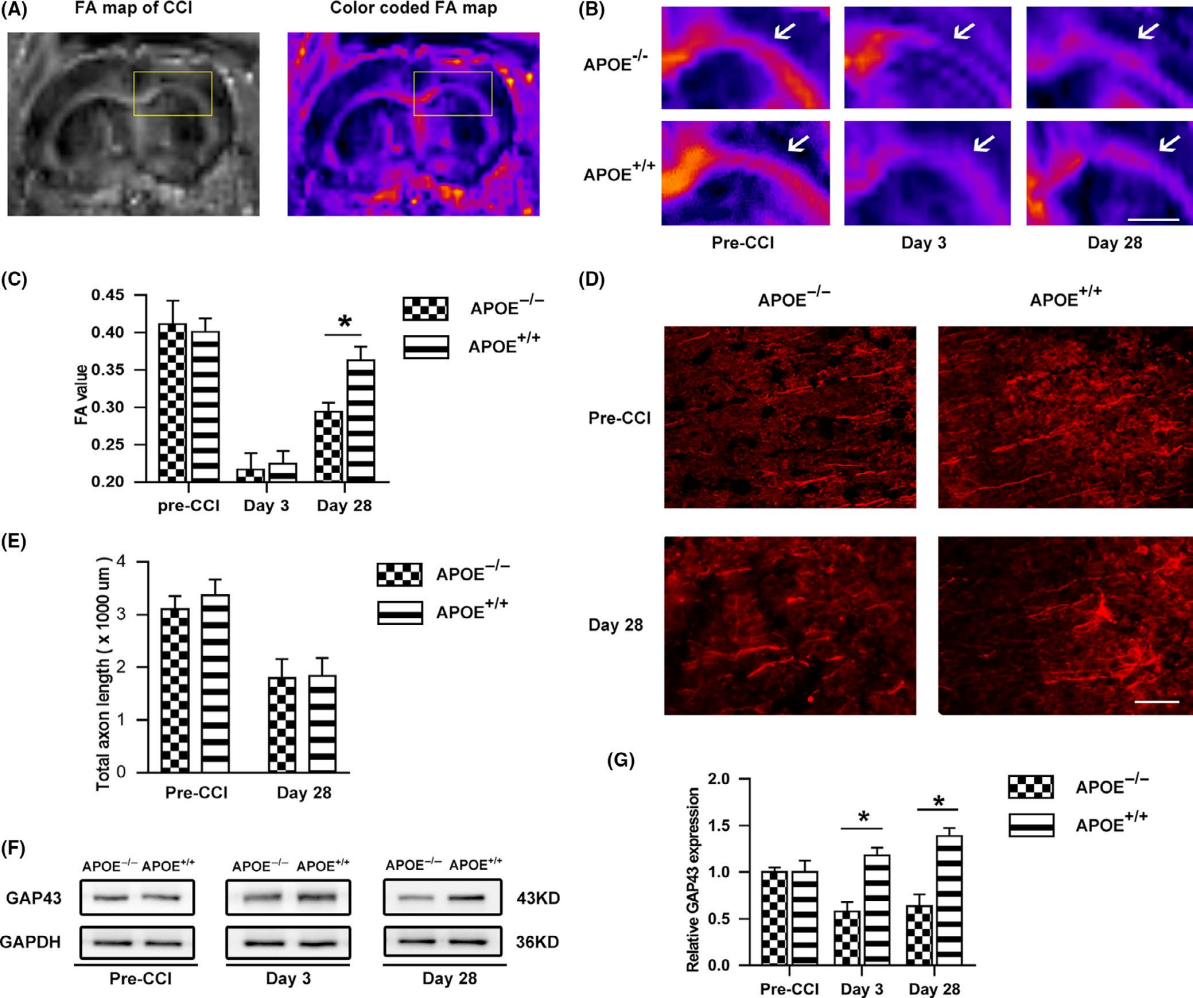
Effects of ApoE on axon remodeling in white matter and gray matter following CCI. (A) Illustrative images of white matter tracts with DTI. The box represents the ROI of the corpus callosum (CC). The original gray scale FA map was color coded by ImageJ so that the CC could be clearly visible. Scale bar = 5 mm. (B) Representative images and (C) quantification of ipsilateral FA in the CC before and after CCI. White arrows point to the CC. Before injury, no difference was detected in the FA value between APOE^−/−^ and APOE^+/+^ mice. After injury, in the early phase (3 days postinjury), there was no significant difference between the groups (*P > *.05), whereas the APOE^+/+^ group exhibited significantly higher FA values than the APOE^−/−^ group at 28 days postinjury. Scale bar = 5 mm. (D) Representative images and (E) quantification of axon length in the ipsilateral pericontusional cortex before and after CCI. Axons in gray matter were stained with SMI‐32. No difference was detected between the two groups in total axon length. Scale bar = 100 µm. (F) Representative bands and (G) quantitative analysis of GAP43 before injury and at 3 days or 28 days after CCI. Compared with the level in the APOE^+/+^ group, the GAP43 level was significantly decreased in the APOE^−/−^ group at both 3 and 28 days postinjury (*P* < .05). N = 6/group. *, *P* < .05

ParaVision 6.0 (Bruker BioSpin) was used for all image analyses. Regions of interest (ROIs) in the CC were carefully defined by anatomical boundaries, with the anterior boundary being the first section containing the hippocampus and the posterior boundary being the last section in which the CC crosses at the midline. This definition resulted in a total of three slices per mouse. The signal intensity within each ROI was measured across slices and weighted by the number of voxels in each sketched region to obtain FA measures. The corpus callosum was included in the white matter ROI with the ventral boundary being drawn at the interface between the hippocampus and thalamus (Figure 1A, red outline). The gray scale FA map was color coded by ImageJ software. The FA map that included the most region of a complete CC was chosen as a representative image. N = 6 for each group.

### 2.4 Immunofluorescence

Mice were deeply anesthetized and fixed for transcardial perfusion fixation with 0.1 M phosphate‐buffered saline (PBS), followed by 4% paraformaldehyde. Brains were removed and cut into 20‐µm serial coronal cryostat sections. Brain slices were air‐dried, fixed in 4% paraformaldehyde, and then blocked in 5% fetal bovine serum for 80 min. After incubation overnight at 4°C with primary antibodies, including SMI‐32 (1:200, Calbiochem), synaptophysin (SYN; 1:200, Millipore), glutathione S‐transferase (GST)‐π (1:400, MBL), β‐amyloid precursor protein (APP) (1:100, Abcam), and platelet‐derived growth factor receptor (PDGFR)‐α (1:100, R&D Systems), slices were washed with PBS and incubated with secondary antibodies. Final staining was observed using a fluorescence microscope (DM6000, Leica). For axon length measurement, the total length of SMI‐32 was averaged from four randomized 20X fields in the pericontusional cortex. For synaptophysin quantification, the immunoreactivity of synaptophysin surrounding the injured area was determined in four randomized 20X fields (two in the cortex and two in the subcortex). For APP quantification, the immunoreactivity of APP was measured in four randomized 20X fields in the corpus callosum. For oligodendrocyte precursor cell (OPC) and oligodendrocyte (OC) quantification, the number of PDGFR‐α‐positive cells (OPC) and GST‐π‐positive cells (OC) was counted in four randomized 20X fields in the corpus callosum. N = 6 for each group.

### 2.5 Behavioral tests

For all behavioral testing, experimenters were blinded to injury, genotype status, and experimental conditions. N = 10 for each group.

Neurological severity score (NSS) test: Before and after the CCI model (one day preinjury and day 1, day 3, day 7, day 14, and day 28 postinjury), the neurological functions of the mice were evaluated according to a set of neurobehavioral tasks to determine the NSS. A 10‐point NSS was applied. The score consisted of scores on 10 different tasks evaluating motor ability, alertness, balance, and general behavior. One point was awarded for failure to complete a task.

Rotarod test: Motor impairments were examined on a rotarod instrument (ZB‐200, Rota‐Rod Instrument). Animals were pretrained for three consecutive days before CCI and then tested at one day preinjury and day 1, day 3, day 7, day 14, and day 28 postinjury. The trial ended when the mouse fell from the rod or 5 minutes had elapsed. The data are presented as latency to fall in seconds and normalized to the preinjury values.

Morris water maze (MWM): The MWM was used to evaluate cognitive function post‐CCI beginning at 15 days postinjury to allow for the recovery of motor deficits. The apparatus consisted of a white pool 150 cm in diameter and 40 cm deep (ZS‐001 Morris). Visible cues were positioned on the walls of the tank and around the room. A goal platform 8 cm in diameter was positioned 1 cm below the surface of the water (hidden platform). After five sets of hidden platform trials, two sets of visible platform trials were performed. The maximum time allotted to reach the platform was 90 s. Performance in the hidden and visible platform trials was quantified as latency to reach the platform in seconds.

Y maze: Spontaneous alternation behavior, as an index of attention, was evaluated by the Y maze test. The test was performed using the reported procedure and was conducted between 9 AM and 4 PM at 28 days postinjury. The mice were moved into the behavioral room for at least 1 hour before testing. The experiment was performed at 35 lux. Before the behavioral test, the mice were placed in one of the compartments and allowed to move freely in one of the arms for 10 min. Each mouse performed one trial. An arm entry was defined as three legs entering one of the arms.

### 2.6 Western blot analysis

For the in vivo study, mice from each group were sacrificed, brain tissues were obtained, and the ipsilateral hemisphere was collected and lysed. For the in vitro study, neuron cultures from each group were washed with 1% ice‐cold PBS containing 10 mM NaF (Sigma Aldrich,) and harvested in ice‐cold lysis buffer (50 mM Tris‐HCl, pH 8.0, 0.5 M NaCl, 0.1% Triton X‐100, protease inhibitor, phosphatase inhibitor cocktails). After sonication, lysates were cleared by centrifugation at 20 000 *g* for 20 minutes at 4°C. Proteins were collected, mixed with SDS‐PAGE loading buffer, boiled, separated in a 10% polyacrylamide gel, and transferred to PVDF membranes. Membranes were blocked and then incubated with primary antibodies. Secondary antibodies coupled to horseradish peroxidase were subsequently applied. Primary antibodies targeting the following proteins were used: Dab1 (1:200, Santa Cruz, USA), phosphorylated Dab1 (pDab1; Tyr 232) (1:200, Santa Cruz), Cdc42 (1:500, Cell Signaling, USA), growth‐associated protein 43 (GAP43) (1:500, Abcam), SYN (1:1000, Millipore), postsynaptic density protein 95 (PSD‐95) (1:1000, Cell Signaling), myelin basic protein (MBP) (1:500, Abcam), and glyceraldehyde‐3‐phosphate dehydrogenase (GAPDH) (1:1000, Abcam). ECL reagents (Beyotime) were used to detect antibody labeling. The results of Western blotting were analyzed by Quantity One software. N = 6 for each group.

### 2.7 Pull‐down assay

The Cdc42 activation assay was performed according to the manufacturer's protocols (Cytoskeleton). After lysis, equivalent protein amounts of lysates were incubated with RAK‐RBD affinity beads for 1 h at 4°C on a rotator. The 2X Laemmli sample buffer was added to resuspend the bead sample. After boiling, the protein sample was ready for Western blot examination. N = 6 for each group.

### 2.8 Primary cortical neuron culture

Brains were harvested by a standard enzyme treatment protocol from neonatal mice born within the previous 24 h. The cerebral cortex was minced into 2 mm^3^ pieces and dissociated with trypsin to obtain a cell suspension. After centrifugation, the resulting cell pellets were resuspended in DMEM/F12 containing 10% fetal bovine serum (FBS) and added to the culture. Primary cultures were maintained in a humidified incubator with 5% CO_2_ and 95% air at 37°C. The culture medium was replaced with fresh medium every two days.

The mechanical injury model was employed as previously described to mimic TBI in vitro.^14^ Prior to scratch injury, Dab1 shRNA lentivirus was used to inhibit Dab1 expression, while NC shRNA lentivirus was applied as a control. Each of these viruses was diluted to 2 × 10^6^ pfu/mL to infect primary neurons for 48 h. After culturing for 5 days in dishes, neurons were dissected using a 10‐µL pipette tip, producing an approximately 0.5‐mm‐wide linear injury across the culture plates. Immediately after the scrape, the medium in the plates was replaced with fresh medium and returned to the incubator. Following injury, recombinant mouse ApoE protein (10 μg/mL in PBS, Peprotech) or BSA protein (10 μg/mL in PBS, Abcam) was added to the culture medium.

### 2.9 Immunocytochemistry

Cultured cells were fixed with 4% paraformaldehyde, followed by incubation with 0.1% Triton X‐100. Cell slides were incubated with anti‐βIII‐tubulin (1:500, Sigma) primary antibodies. The resulting slides were observed through a fluorescence microscope (DM6000, Leica), and images were captured with the Leica Application Suite. For growth cone analysis, the same immunocytochemical protocol for ApoE (1:200, Santa Cruz) was followed, plus phalloidin (1:100, Sigma) incubation before laser confocal microscopy (Leica Microsystem) scanning. The images were captured with Nikon Elements Imaging software.

To analyze axonal regeneration, we assessed the total axon length of βIII‐tubulin‐positive axons crossing the scratched blank regions with ImageJ software (NIH). To assess growth cone formation, we determined the average area per growth cone and the number of growth cones per image area in the lesion gap. Ten images from five separate wells were randomly measured in each experimental group. The growth cone area was determined by drawing a polygon around the palm of the growth cone, followed by area measurement using ImageJ. N = 6 for each group.

### 2.10 Statistical analysis

The results are represented as the mean ± standard deviation (SD). Statistical analysis was performed using Prism version 5 (GraphPad). Motor and MWM test data from the APOE‐deficient mice and Dab1 shRNA interference mice were compared to data from APOE^+/+^ controls using two‐factor repeated‐measures analysis of variance (ANOVA; for group and time). The remaining data were analyzed using Student's t test or randomized one‐way ANOVA, followed by Tukey's post hoc test, to compare the differences between groups. Differences were considered statistically significant at *P* < .05.

## 3 RESULTS

### 3.1 APOE deficiency inhibits axonal regeneration following CCI

To investigate the effects of ApoE protein on white matter integrity post‐CCI, we performed MRI examination on experimental animals at different time points. The FA parameter has been demonstrated to be a sensitive indicator of white matter integrity, which is positively correlated with regenerated axon number and diameter. Under normal conditions, no difference was detected in the FA value between APOE^−/−^ and APOE^+/+^ mice. After CCI, separate cohorts of mice were scanned at 3 and 28 days, representing the acute injury phase and late recovery phase, respectively. As shown in Figure 1B,C, in the early phase (3 days postinjury), there was no significant difference between groups, whereas the postinjury APOE^+/+^ group exhibited significantly higher FA values than the APOE^−/−^ group (*P < *.05) at 28 days postinjury.

Subsequently, the total axon length in gray matter at the pericontusional cortex was compared between groups, as determined by SMI‐32 immunoreactivity. Before injury, the intact axons were arranged in parallel, whereas CCI greatly disrupted the axon structure in the pericontusional cortex. No difference was detected between the two groups in total axon length before and after CCI (Figure 1D,E).

To better quantify axonal regeneration in vivo, the level of GAP43 in the ipsilateral hemisphere was measured by Western blot. As shown in Figure 1F,G, the GAP43 level was significantly lower in the APOE^−/−^ group than in the APOE^+/+^ at both 3 and 28 days postinjury (*P < *.05).

### 3.2 Alterations in related axonopathy by ApoE following CCI

In addition to axonal restoration, the potential effects of ApoE protein on synaptogenesis, myelination, and axonal injury were further assessed. For synaptogenesis assessment, we first examined the expression levels of PSD95 and synaptophysin by Western blot and determined the immunoreactivity of synaptophysin surrounding the injured area. For axonal injury assessment, we measured the expression of APP in the corpus callosum at the early stage. For remyelination assessment, we counted the number of PDGFR‐α‐positive cells (OPCs) at 7 days postinjury and GST‐π‐positive cells (OCs) at 28 days postinjury at the corpus callosum.

**Figure 2:**
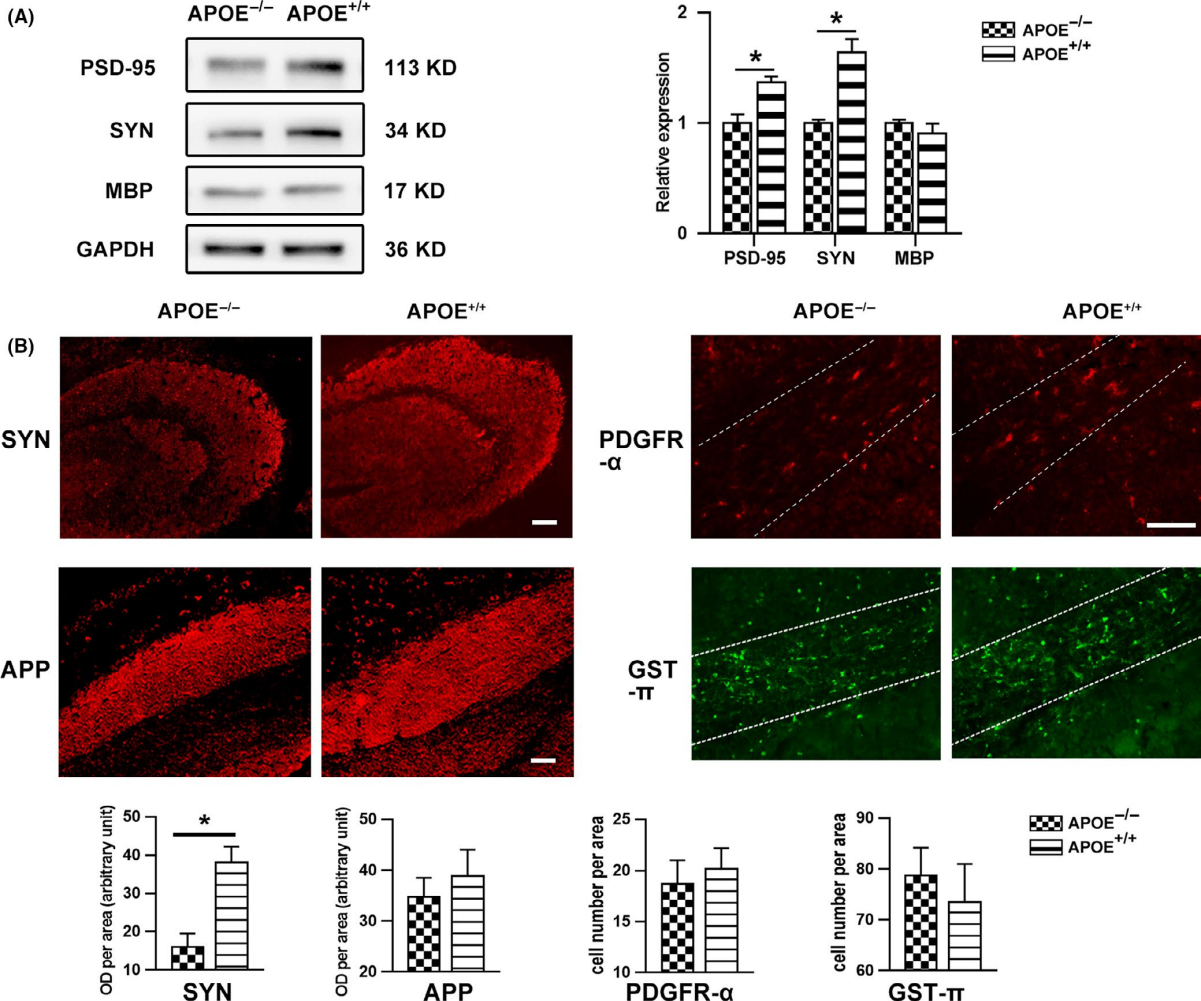
Alteration of axonopathy by ApoE following CCI. (A) Representative bands and quantitative analysis of synaptophysin (SYN), PSD‐95 and MBP expression at 28 days after CCI. The data were normalized to those of the APOE^−/−^ group. MBP expression levels did not differ between the APOE^−/−^ and APOE^+/+^ groups (*P* > .05), while APOE deficiency significantly suppressed SYN and PSD‐95 expression (*P* < .05). (B) Representative images and quantitation of SYN (28 days), APP (3 days), PDGFR‐α (7 days), and GST‐π (28 days) immunoreactivity. The number of PDGFR‐α‐positive cells or GST‐π‐positive cells in corpus callosum (outlined by white dotted line) was calculated for oligodendrocyte precursor cell (OPC) and oligodendrocyte (OC) quantification, respectively. SYN intensity in the APOE^−/−^ neurons was lower than that in the APOE^+/+^ neurons (*P < .*05), while no differences were detected in APP intensity or PDGFR‐α‐positive cell (OPC) or GST‐π‐positive cell (OC) number. Scale bar = 100 µm. N = 6/group. *, *P* < .05

In the Western blot analysis (Figure 2A), MBP expression did not differ between the APOE^−/−^ and APOE^+/+^ groups (*P > .*05); however, APOE deficiency significantly suppressed synaptophysin and PSD‐95 expression (*P < *.05). Accordingly, the immunofluorescence results (Figure 2B) showed lower synaptophysin intensity in APOE‐deficient mice than in APOE^+/+^ mice following CCI (*P* < .05) but no differences in APP intensity, PDGFR‐α‐positive cell (OPC) number or GST‐π‐positive (OC) cell number (*P* > .05).

### 3.3 ApoE improves neurobehavioral function following CCI

**Figure 3:**
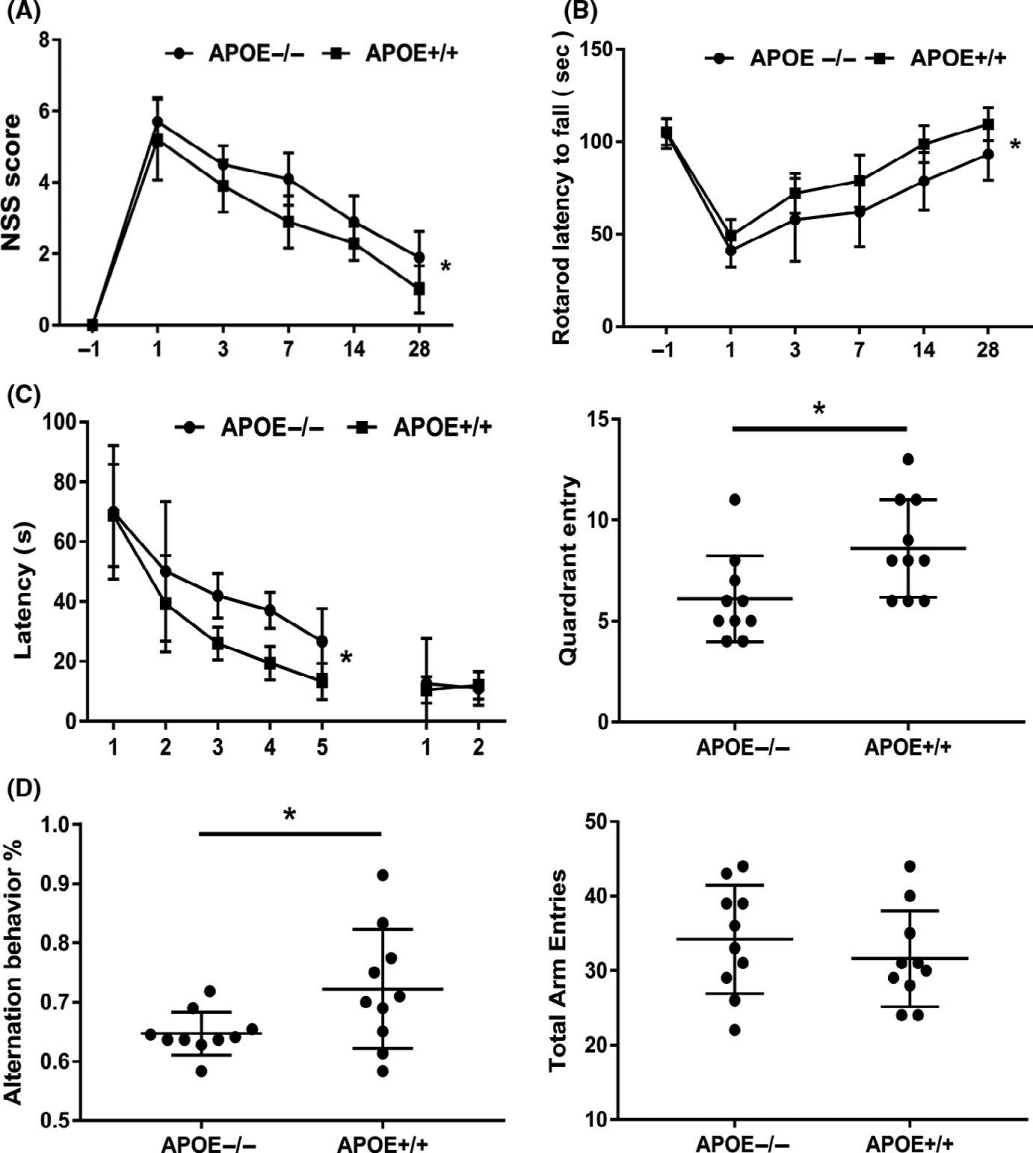
Effects of ApoE on motor and cognitive function following CCI. (A) NSS and (B) rotarod latency 1 day preinjury and 1, 3, 7, 14, and 28 days after CCI. The NSS and rotarod test were applied to evaluate the motor function of mice after injury. Motor function was markedly impaired by CCI. Notably, the APOE^−/−^ group displayed overall impairments compared to the APOE^+/+^ group in both the NSS and rotarod tests. (C, D) Latency and probe trials in the MWM from 15 days post‐CCI. (E, F) Y maze test at 28 days after CCI. The MWM and Y maze tests were applied to evaluate the cognitive function of mice after injury. During the learning trials in the water maze, APOE^+/+^ mice showed an overall advantage in cognitive performance compared to APOE^−/−^ mice following CCI (C). At the end of training, spatial memory was tested by target quadrant entries in each group, and APOE^+/+^ mice were more inclined to enter the target quadrant than the other groups (D). Likewise, APOE^−/−^ mice showed a significant decrease in alternation behavior in the Y maze compared to the APOE^+/+^ group (E). However, there was no difference in total arm entries between the groups (F). N = 10/group. *, *P < *.05

To evaluate the potential benefits of ApoE‐induced axonal regeneration, we conducted a series of behavioral tests in APOE^−/−^ and APOE^+/+^ mice, that is, the NSS, rotarod, MWM, and Y maze tests. As shown in Figure 3A,B, motor function was markedly impaired by CCI. Notably, repeated‐measures analysis revealed that the APOE^−/−^ group displayed overall greater impairments in both the NSS and rotarod tests compared to the APOE^+/+^ group (*P < *.05).

In addition, cognitive function after CCI was evaluated by the MWM (Figure 3C,D) and Y maze tests (Figure 3E,F). During the learning trials in the water maze, APOE^+/+^ mice showed an overall advantage in cognitive performance over APOE^−/−^ mice following CCI by ANOVA analysis (*P < *.05). At the end of training, spatial memory was assessed by the number of target quadrant entries in each group, showing that the APOE^+/+^ mice were more inclined to enter the target quadrant than APOE^−/−^ mice (*P < *.05). Likewise, APOE^−/−^ mice showed significantly less alternation behavior in the Y maze than the APOE^+/+^ group at 28 days post‐CCI (*P < *.05). However, there was no difference in total arm entries between the groups (*P > *.05).

### 3.4 The Dab1‐dependent pathway is activated and has a beneficial effect in ApoE‐induced axonal regeneration following CCI

To study whether the Dab1‐Cdc42 pathway regulates the effects of ApoE on the reconstruction of the neural network after acute brain injury, we used Western blotting to detect changes in pathway regulators and the downstream target GAP43.

**Figure 4:**
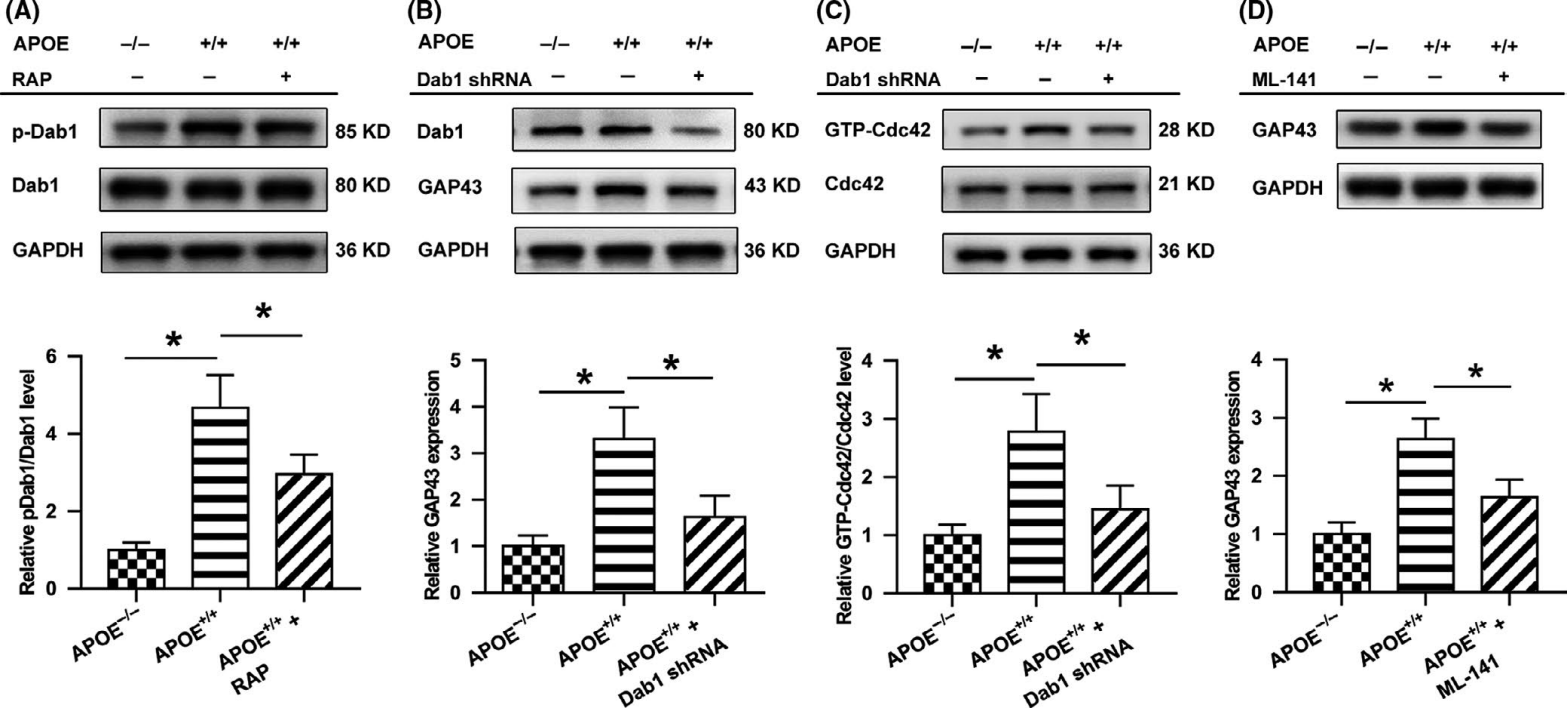
ApoE induces the activation of Dab1 and Cdc42 after CCI. (A) Representative bands and quantitative analysis of the ratio of phosphorylated Dab1(pDab1) at 3 days after brain injury. Dab1 phosphorylation was significantly suppressed in the APOE^−/−^ group compared to that in the APOE^+/+^ group, whereas RAP, a competitive receptor blocker known to strongly inhibit ligand binding to ApoE receptors, abolished this difference. (B) The level of Dab1 in APOE^+/+^ mice was suppressed by Dab1 shRNA lentivirus in the APOE^+/+^+Dab1 shRNA group. Following injury, a higher level of GAP43 was observed in the APOE^+/+^ group than in the APOE^−/−^ group. However, GAP43 levels were lower in the APOE^+/+^+Dab1 shRNA group than in the APOE^+/+^ group. (C) The ratio of GTP‐Cdc42/Cdc42 at 3 days following brain injury was measured. The level of activated Cdc42 was significantly higher in the APOE^+/+^ group than in the APOE^−/−^ group. However, compared with the level in the APOE^+/+^ group, activated Cdc42 was lower in the APOE^+/+^+Dab1 shRNA group, in which Dab1 was downregulated. (D) ML141 (a Cdc42 activation inhibitor) treatment showed a similar effect as that of Dab1 shRNA. GAP43 levels were lower in the ML141 treatment group than in the APOE^+/+^ group. The expression levels of each protein were normalized to those in the APOE^−/−^ group. N = 6/group. *, *P < *.05

**Figure 5:**
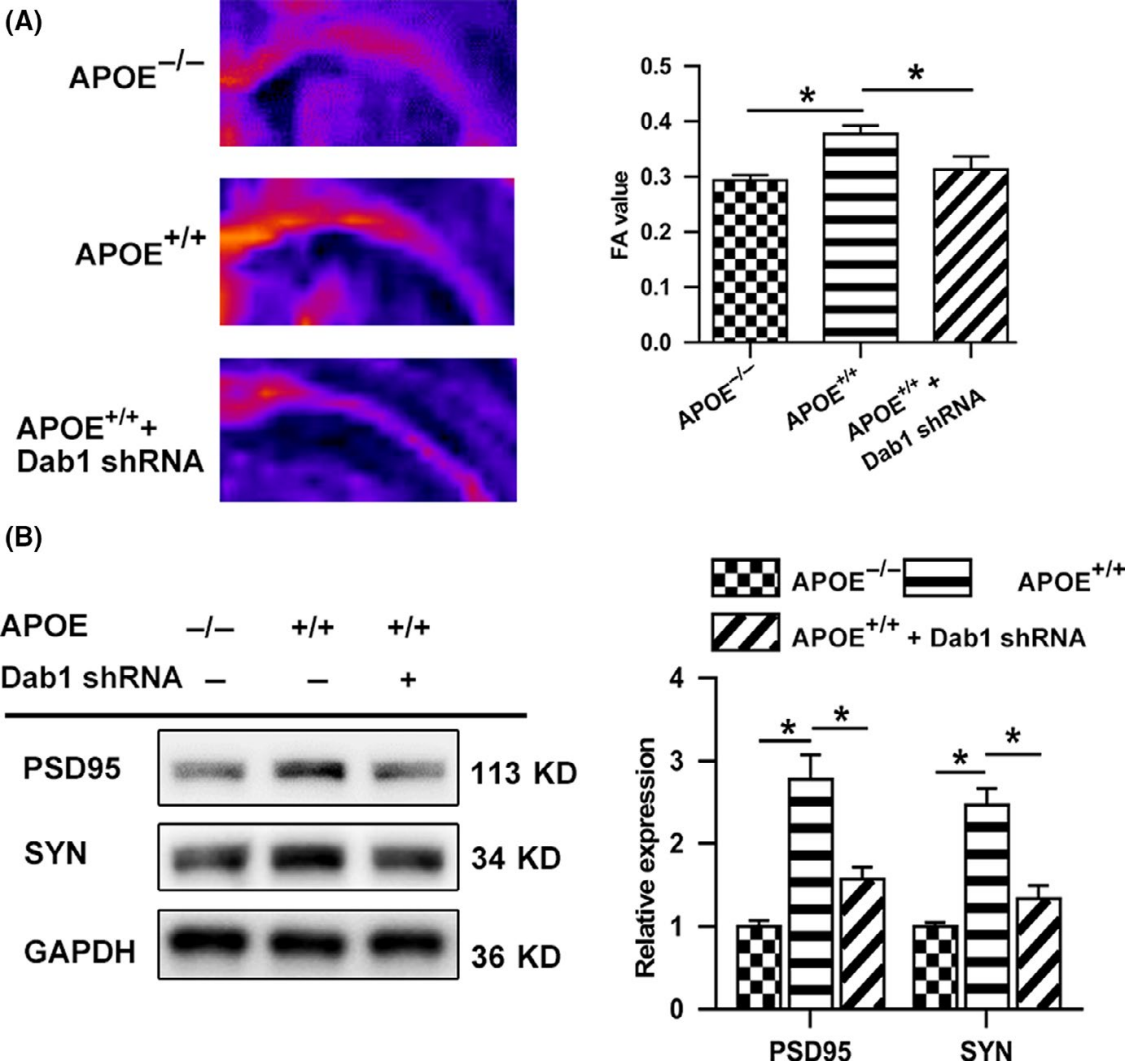
Effects of Dab1 on white matter integrity and axon regeneration following CCI. (A) Representative images and the quantification of ipsilateral FA in the CC before CCI and at 28 days after CCI. Scale bar = 5 mm. (B) Representative bands and quantitative analysis of SYN and PSD‐95 in the lesion boundary zone at 28 days postinjury. Compared to the APOE^−/−^ group, the APOE^+/+^ group exhibited significantly higher FA values at 28 days postinjury, as well as increased expression of SYN and PSD‐95 (*P < *.05). Compared to the APOE^+/+^ group, the APOE^+/+^+Dab1 shRNA group exhibited significantly lower FA values at 28 days postinjury and reduced SYN and PSD‐95 expression. The data were normalized to those in the APOE^−/−^ group. N = 6/group. *, *P < *.05

As shown in Figure 4A, Dab1 phosphorylation was significantly suppressed in the APOE^−/−^ group compared to that in the APOE^+/+^ group (*P* < .05), whereas RAP, a competitive receptor blocker that strongly inhibits ligand binding to ApoE receptors, abolished this effect of ApoE (*P* < .05). Following injury, a higher level of GAP43 was observed in the APOE^+/+^ group than in the APOE^−/−^ group (*P* < .05), but this difference in expression was inhibited by Dab1 shRNA (Figure 4B) and ML141 (a Cdc42 activation inhibitor) treatment (Figure 4D) (*P* < .05). Likewise, the GTP‐Cdc42/Cdc42 ratio was significantly higher in the APOE^+/+^ group than in the APOE^−/−^ group (*P* < .05); however, this ratio decreased when APOE^+/+^ animals were treated with Dab1 shRNA (*P* < .05), as shown in Figure 4C. Moreover, Dab1 inhibition abolished the beneficial effects of ApoE on white matter integrity (Figure 5A) and synaptogenesis (Figure 5B) (*P* < .05).

### 3.5 Dab1 inhibition reverses ApoE‐induced functional recovery following CCI

**Figure 6:**
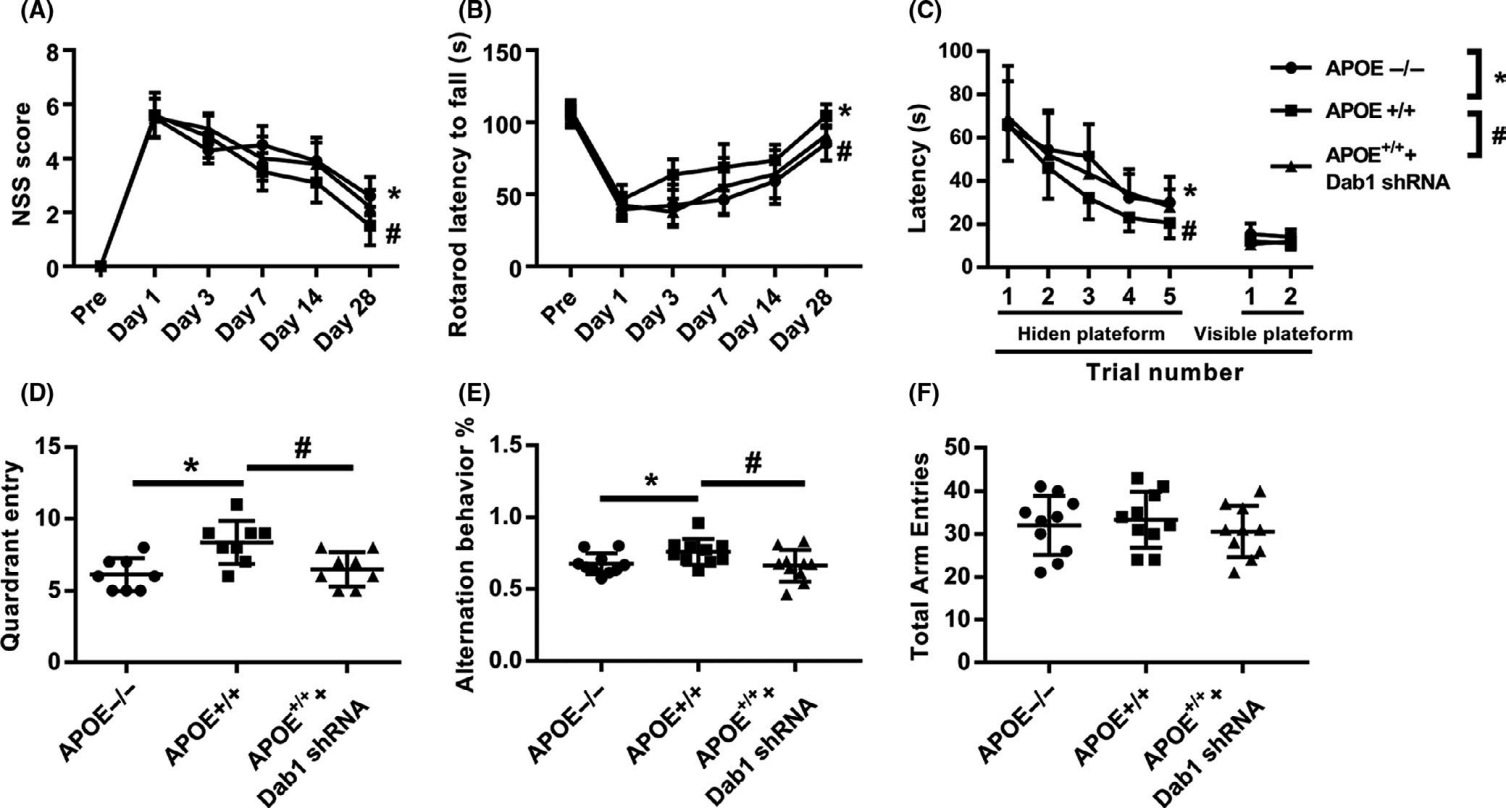
Effects of Dab1 on motor and cognitive function following CCI. (A) NSS and (B) rotarod latency to fall at 1 day preinjury and 1, 3, 7, 14, and 28 days after CCI. (C, D) Latency and probe trials in the MWM from 15 days post‐CCI. (E, F) Y maze test at 28 days after CCI. Consistent with previous experiments, APOE^+/+^ mice showed significantly better performance than APOE^−/−^ mice in terms of motor functions (A‐B) and cognitive functions (C‐F) (*P < *.05). However, Dab1 shRNA pretreatment sufficiently abolished the protective effects of APOE, as shown in these tests. *, APOE^−/−^ compared with APOE^+/+^, *P < *.05. #, APOE^+/+^+Dab1 shRNA compared with APOE^+/+^, *P* < .05. N = 10/group

To validate the role of Dab1 in functional recovery after TBI, we repeated the behavioral tests in APOE^−/−^ and APOE^+/+^ mice (Figure 6). Consistent with previous experiments, APOE^+/+^ mice showed significantly better motor function (Figure 6A,B) and cognitive function (Figure 6C‐F) than APOE^−/−^ mice (*P < *.05). However, Dab1 shRNA pretreatment sufficiently abolished the protective effects of ApoE, as shown in these tests (*P < *.05).

### 3.6 ApoE improves residual axon extension and growth cone formation following mechanical neuronal injury

To clarify the potential spatial association between ApoE and axons, we double‐stained cultured primary cortical neurons with F‐actin and ApoE (Figure S1). ApoE colocalized with the F‐actin‐enriched growth cone before and after axotomy, indicating that ApoE may play a role in growth cone formation.

**Figure 7:**
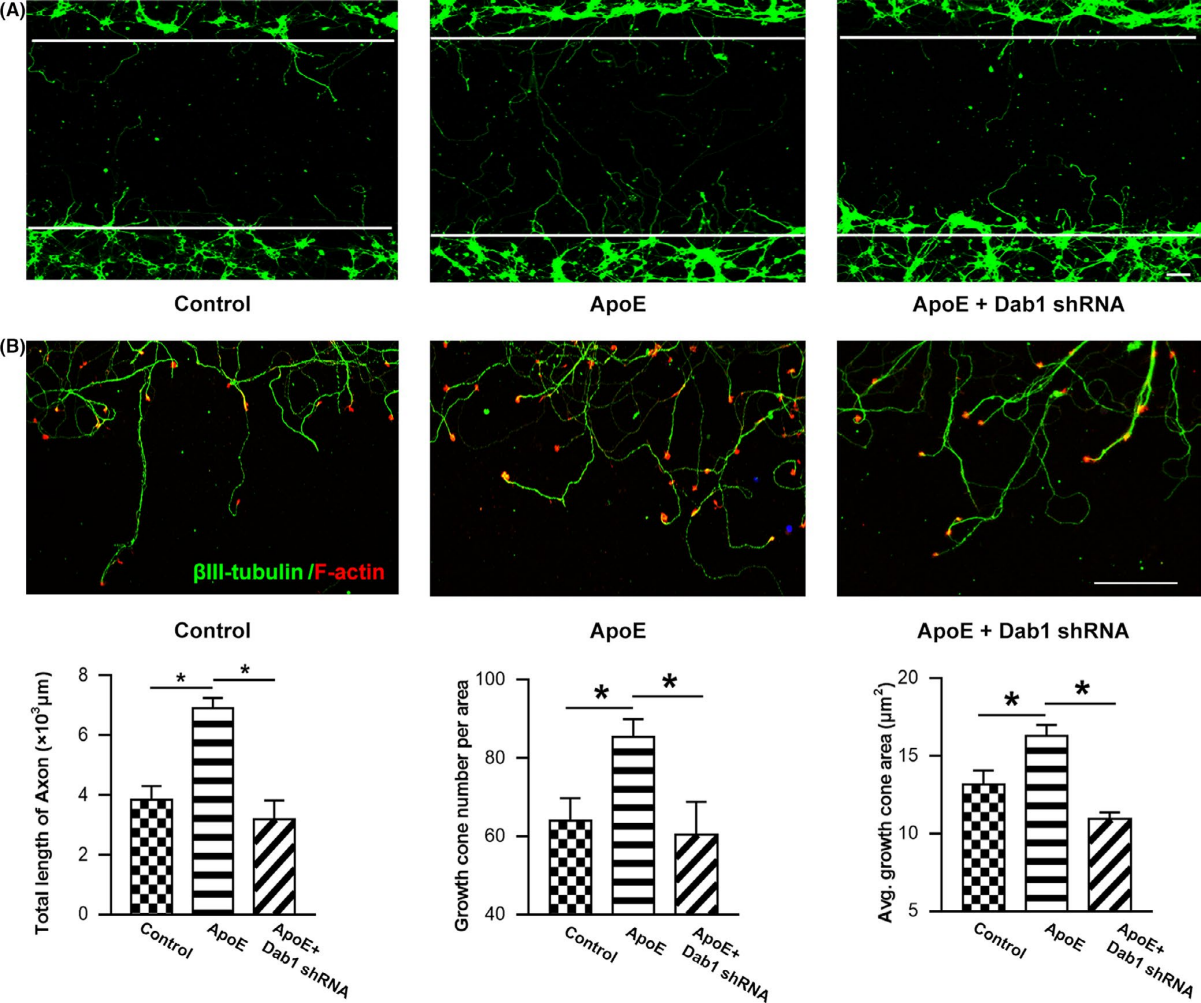
ApoE improves axonal outgrowth and growth cone formation in injured neurons. Axons were stained at 24 h after mechanical injury. (A) Representative images of axonal outgrowth after scratch injury. βIII‐tubulin‐positive neurites (green) that crossed over the white line margin were measured to quantify the regenerated axonal length. Scale bar = 200 µm. ApoE treatment induced significant elongation in total axonal length compared to vehicle control treatment. However, compared to the ApoE treatment group, the Dab1 shRNA pretreatment group exhibited an attenuation of total regenerated axonal length due to Dab1 expression downregulation (*P < *.05). (B) Representative images of growth cone regeneration. Phalloidin staining (red) showed growth cones at the tips of regenerating βIII‐tubulin‐positive neurites (green). Scale bar = 50 µm. The number of growth cones visible per area in the lesion gap and the average growth cone area were quantified. Along with the regenerated axonal extension, the number of growth cones was significantly higher after ApoE treatment than the number in the control group. Likewise, the average area per growth cone in the ApoE‐treated group was significantly larger than that in the control group (*P < *.05). However, the effects of ApoE were abolished when Dab1 was downregulated by pretreatment with Dab1 shRNA. *P < .05. n = 6/group

To verify axonal outgrowth after TBI, we created a scratch injury in culture. As shown in Figure S1, the lesion area was almost devoid of cell bodies and neurites immediately after injury. Then, regenerated axons gradually extended into the lesion area, peaking at 24 h postinjury. Axonal extensions were quantified by total axon length in the lesion gap at 24 h postinjury. As shown in Figure 7A, ApoE treatment induced significant elongation in total axonal length compared to vehicle control treatment. However, the effect of ApoE was attenuated by Dab1 shRNA.

The potential role of ApoE in growth cone formation was additionally evaluated, including the number and area of growth cones postinjury. Along with axonal extension, newly generated growth cones emerged at the terminals of regenerated axons in the gap area (Figure 7B). As shown, the number of growth cones was significantly elevated after ApoE treatment compared with the number in the control group. Likewise, the average area per growth cone in the ApoE‐treated group was significantly larger than that in the control group. However, such effects of ApoE were abolished in the presence of Dab1 shRNA (*P < *.05).

## 4 DISCUSSION

Following TBI, axonal damage is believed to be responsible for neurological deficits. However, spontaneous outgrowth from the axotomized axon tip has also been observed postinjury. To explore the potential pathway mediating axonal remodeling, we measured white matter tract integrity in CCI mice, as well as growth cone formation and axonal outgrowth following mechanical injury in vitro. The major findings in the current study include the following: (a) ApoE promotes axonal regeneration in vivo and in vitro; (b) ApoE activates the Dab1‐Cdc42 pathway during axonal remodeling; and (c) ApoE contributes to functional recovery via Dab1.

In experimental animals and clinical patients, noninvasive DTI has recently been employed as a novel method for the detection of axonopathy in Alzheimer's disease,^15^ neonatal hypoxia‐ischemia,^16^ multiple sclerosis,^17^ traumatic spinal cord injury,^18^ and TBI.^19^ DTI is a quantitative measurement of water diffusion that provides information regarding the regional directional asymmetry (anisotropy) of white matter. The association between DTI parameters (relative anisotropy [RA], radial diffusivity [RD], FA) and histological axonal changes within pericontusion white matter following TBI has been studied,^20^, ^21^ and the FA value shows the strongest correlation with the regenerated axon number and diameter.^22^ Moreover, the FA value is associated with neurological behavioral performance in hypoxia‐ischemia stroke,^23^ brain radiation injury 22 and TBI.^20^ Hence, the FA parameter has been demonstrated to be a sensitive indicator of white matter integrity. We found that there was no difference in the FA value between the injury groups at 3 days postinjury, indicating that ApoE may exert no effects on acute axonal injury, which is supported by APP staining in white matter. In the regeneration phase, however, APOE‐deficient mice exhibited significantly impaired white matter integrity, as indicated by lower FA values than those in APOE^+/+^ mice. In contrast, axon regeneration in gray matter was unchanged by APOE deficiency, which could be explained by the low baseline axon density in such an area. Due to the working principle of DTI, axonal regeneration is difficult to distinguish from remyelination with FA. To solve this puzzle, we measured markers of remyelination using multiple parameters. After measuring MBP expression, OPC number and OC number, we cautiously concluded that ApoE is efficient in promoting axonal regeneration, rather than remyelination, following TBI.

As a membrane‐anchored intracellular growth‐associated protein predominantly expressed in growth cones during development, GAP43 has been applied as a marker of axonal regeneration.^24^, ^25^ Western blot results indicated that ApoE significantly upregulated GAP43 expression at both 3 and 28 days post‐CCI. This discrepancy in the MRI results implies that axonal regeneration was initiated starting in the early phase and eventually contributes to white matter reconstruction in the late phase. Moreover, the expression of synaptogenesis markers, including synaptophysin and PSD95, was also increased by ApoE. In accordance with the benefits of synaptogenesis, these data support a positive role of ApoE in axonal remodeling following TBI.

In principle, treatment to promote axonal regeneration could be beneficial for functional recovery after brain injury. In survivors of TBI, the most impaired neurological functions are related to motility and cognition; thus, the NSS was assessed and the rotarod test, MWM test, and Y maze test were performed to measure such deficits. Another study on closed head injury also found that APOE‐deficient mice exhibited worse motor and cognitive performance than their wild‐type counterparts, persisting for 40 days or longer after the injury.^26^ Our results show that both motor and cognitive deficits worsened after APOE ablation, suggesting that ApoE acts to improve functional outcomes after TBI.

Until now, the mechanism underlying ApoE‐driven neural restoration has been poorly characterized, partly due to the wide spectrum of ApoE ligand‐receptor interactions. ApoE can bind to many neuronal LDL receptor family members, initiating serial downstream cascades. In this study, we focused on the Dab1 pathway because this adaptor protein was previously reported to bind to the C‐termini of ApoE receptors. ApoE stimulates the phosphorylation of Dab1 by binding with its receptors, such as ApoER2 and VLDLR.^8^, ^27^, ^28^ Dab1 has been well documented to be a critical regulator in the development of neural networks.^29^ Dab1 is enriched in the axonal growth cone, and tyrosine phosphorylation of Dab1 is increased during axon outgrowth 30; in contrast, Dab1 phosphorylation inhibition prevents neuronal dendrite outgrowth.^31^, ^32^ Accordingly, Dab1 downregulation in vivo causes behavioral impairments during development.^33^ These observations raise more questions about whether Dab1 exerts similar effects on the reconstruction of neural networks, especially after acute brain injury. We found that Dab1 phosphorylation was activated in response to ApoE following brain injury. Furthermore, the effects of ApoE on axonal regeneration were attenuated by Dab1 shRNA in vitro and in vivo. As the downstream effector of Dab1, Cdc42 has previously been documented to promote actin accumulation in the growth cone to further potentiate axon elongation.^12^ More importantly, Cdc42 can be activated by pDab1 to promote axonal branching and growth cone motility in cultured primary neurons.^11^ Here, we demonstrated that Cdc42 activity was enhanced in the presence of ApoE, but this enhancement was greatly inhibited by Dab1 shRNA transfection, even in the presence of ApoE. Furthermore, treatment with the Cdc42 inhibitor ML141 inhibited the upregulation of GAP43, which resulted in a similar effect as Dab1 downregulation. Taken together, these results indicate that the Dab1‐Cdc42 pathway mediates ApoE‐induced axonal regeneration following TBI.

Surviving axons must transform their damaged terminals into new growth cones to initiate regeneration.^34^ With the enrichment of dynamic actin, the de novo assembly of the new growth cone is a prerequisite for axonal elongation from the axotomized tip. APOE gene expression was significantly increased in axotomized cortical neurons in a DNA microarray study in which a total of 305 genes were analyzed.^35^ Our previous study demonstrated that mechanical neuronal injury promotes the synthesis of intracellular ApoE within neurons and the uptake of extracellular ApoE.^14^ Here, we showed that ApoE was expressed in both existing and regenerated growth cones, suggesting that ApoE participates in growth cone formation.

There are several limitations in our study. A specific Dab1 activator would allow for a better assessment of the effect of Dab1 pathway on axonal regeneration, but it has yet to be discovered or created. It would be better to compare the effect of ApoE on Dab1 pathway by using a specific Dab1 activator in the future. Secondly, the human APOE gene has 3 polymorphic alleles, e2 (cys112, cys158), e3 (cys112, arg158), and e4 (arg112, arg158). The e4 isoform has been implicated in atherosclerosis, ischemic cerebrovascular disease, impaired cognitive function, and late‐onset Alzheimer's disease.^36^ Several studies have shown that patients with APOE e4 have a poorer outcome after TBI.^37^ In this study, we explored the mechanism of wild‐type murine ApoE on white matter remodeling after TBI; however, whether different ApoE isoform (e2, e3 or e4) has a similar function should be examined in future studies.

## 5 CONCLUSION

Given that a potential neuroprotective effect of ApoE has been previously recognized, the current study extends the benefits of ApoE in neural restoration following TBI via axonal regeneration. In vivo and in vitro results demonstrate that ApoE initiates the phosphorylation of Dab1, which leads to subsequent Cdc42 activation, ultimately contributing to axonal regeneration and functional recovery.

## CONFLICT OF INTEREST

The authors declare no conflicts of interest.

## Data Availability

The data used to support the findings of this study are available from the corresponding author upon request.
