# Supplementary figures and images for: Apolipoprotein E promotes white matter remodeling via the Dab1‐dependent pathway after traumatic brain injury
Source: CNS Neurosci Ther. 2020 Mar 1;26(7):698–710. doi: 10.1111/cns.13298 (PMC7298982; doi:10.1111/cns.13298)

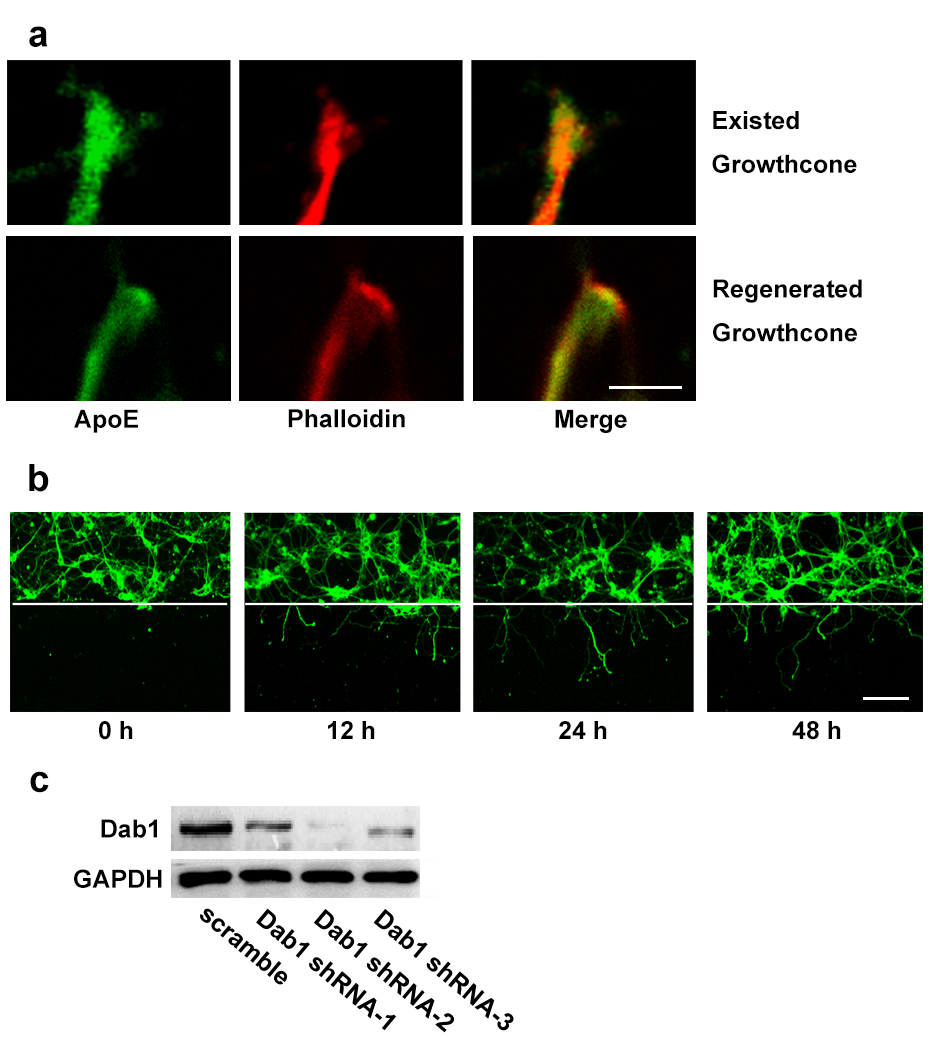

Supplement: Supplementary file 1 [file CNS-26-698-s001.tif]
